# Molecular sexing and preliminary assessment of population sex ratio of the endangered Malayan tapir (*Tapirus indicus*) in Peninsular Malaysia

**DOI:** 10.1038/s41598-020-60552-y

**Published:** 2020-03-04

**Authors:** Qi Luan Lim, Yoeng Leh Tan, Wei Lun Ng, Christina Seok Yien Yong, Ahmad Ismail, Jeffrine J. Rovie-Ryan, Norsyamimi Rosli, Geetha Annavi

**Affiliations:** 1 Department of Biology, Faculty of Science, Universiti Putra Malaysia, Selangor, Malaysia; 2 Wildlife Research Center, Kyoto University, Kyoto, Japan; 3 China-ASEAN College of Marine Sciences, Xiamen University Malaysia, Selangor, Malaysia; 4 National Wildlife Forensic Laboratory, Ex-Situ Conservation Division, Department of Wildlife and National Parks, Kuala Lumpur, Malaysia; 5 Institute of Tropical Biodiversity and Sustainable Development, Universiti Malaysia Terengganu, Terengganu, Malaysia

**Keywords:** Conservation biology, Genetic markers

## Abstract

A molecular sexing method by polymerase chain reaction (PCR) amplification of a portion of the sex-determining region Y (SRY) and the zinc finger (ZF) gene, as well as six equine Y-chromosome-specific microsatellite markers, were tested in the Malayan tapir (*Tapirus indicus*). While the microsatellite markers did not yield any male-specific amplicons for sex-typing, the SRY/ZF marker system produced reliable molecular sexing results by accurately sex-typing 31 reference Malayan tapirs, using whole blood, dried blood spot (DBS), or tissue samples as materials for DNA extraction. The marker system was also tested on 16 faecal samples, and the results were in general consistent with the pre-determined sexes of the animals, despite some amplification failures. A preliminary estimation of wild Malayan tapir population sex ratio was estimated from the Wildlife Genomic Resource Bank (WGRB) database of the Malaysian Department of Wildlife and National Parks (PERHILITAN), zoos, and the Sungai Dusun Wildlife Conservation Centre (WCC), as well as from the results of molecular sexing 12 samples of unknown sex. The overall sex ratio favoured females, but the deviation from parity was statistically not significant when tested using the binomial test (*p* > 0.05), which may be due to reduced statistical power caused by small sample sizes.

## Introduction

Sex ratio, conventionally defined according to life-history stages such as pre-birth (e.g. sex ratio at fertilisation) or post-birth (e.g. sex ratio at birth, adult sex ratio), is used to describe a population^1^. The sex ratio at different stages of life is influenced by one or a set of sex-differential processes such as sex determination, survival, development, maturation, migration, mate competition, and parental investment^2–4^. One of the types, the adult sex ratio, is estimated by counting the number of males and females who have matured, regardless of whether they are sexually active or have produced offspring^2^.

Adult sex ratio can vary depending on species, for examples, in species with heterogametic males (e.g. mammals), the ratio tend to be female-biased, while in species with heterogametic females (e.g. birds), the ratio tends to be male-biased^5^. In mammals, at birth, the populations are expected to have a 1:1 ratio for male to female offspring because of the Mendelian mode of inheritance of sex chromosomes—theoretically half of the offspring would possess the XY genotype, thus developing into males; and if the cost to produce either sex is equal, parity in adult sex ratio is expected^4,6^. However, in reality, mammalian population sex ratios may vary with the costs and benefits of producing male and female offspring^7^.

The Malayan tapir (*Tapirus indicus*) is an endangered mammal species listed in the International Union for Conservation of Nature (IUCN) Red List^8^. This tapir species is the only Old-World species in the family Tapiridae and can be found in regions of Southeast Asia^9^. Its worldwide population is declining due to threats such as habitat loss and fragmentation, poaching, and accidental killings (e.g. road-kill)^8,10–13^. In Peninsular Malaysia where forests are becoming fragmented, there appears to be an increasing trend of displacement—92 tapirs were recorded to be road-killed from year 2006 to 2017^10,14^. The tapir population in Peninsular Malaysia is estimated at 1,100–1,500 individuals^11^, but the actual number could be lower; only 3–5 tapirs were estimated to be present in an isolated forest during a survey in year 2010^15^. This has raised concerns on the sustainability of the tapir populations in isolated habitats because endangered species with small population sizes have a higher risk of extinction due to the effects of anthropogenic^16^ or stochastic factors^17^ such as unsustainable population sex ratios.

The effects of demographic stochasticity and genetic drift, or other genetic factors (e.g. inbreeding depression, reduced genetic variability, accumulation of deleterious mutations) in small populations can affect the rate of reproduction and expansion in the populations and even lead to extinction when coupled with skewed sex ratios^18,19^. In the case of the Malayan tapirs, which are thought to be monogamous or facultative polygynous creatures, an increase in males led to a reduction in stochastic population growth rate in VORTEX (a simulation model for population viability analysis) under the assumption of a monogamous mating system^20^. Therefore, a male-biased sex ratio is an issue in the conservation and management of Malayan tapir populations.

Identifying wild populations with potentially deteriorating skewed sex ratio may help in conservation interventions (i.e. reintroduction or translocation of displaced tapirs from one forest to another) or in the formation of better management plans that address the immediate needs of the Malayan tapirs in isolated habitats. However, little is known about their sex ratio in wild populations. Malayan tapirs only exhibit minor sexual dimorphism in terms of size—females are larger than males—which limits the use of photos and videos obtained from camera traps for sexing^21^. Besides, tapirs have a wide home range, are rare, and hard to capture, making direct observation difficult. As such, a genetic approach to determine the sex ratio of wild populations through non-invasive sampling methods (i.e. through hair, scat, faeces, etc.) presents an alternative method to traditional field observations or camera trapping for the purpose of measuring such population parameters^22^. To initiate such a study, a reliable sex marker would be needed.

Molecular sexing relies on genetic differences on the sex chromosomes. The two DNA regions commonly targeted for sexing of mammals are the sex-determining region Y (SRY) and the zinc finger (ZF) gene^23–25^. The SRY region is situated on the Y chromosome of males^26^; the ZF gene is situated on the X chromosome (ZFX) and the pseudo-autosomal region of the Y chromosome (ZFY), and so is present in both males and females^27^. Depending on the species and method used, the ZF gene may act as a sex-typing marker or as an internal positive control for polymerase chain reaction (PCR)^28,29^. The use of SRY as the main sex-typing marker and ZF as the positive control for PCR amplification success has been successfully demonstrated in the lowland tapir (*Tapirus terrestris*), where the presence of an SRY amplicon denotes a male^25^. Other Y-chromosome segments such as Y-linked microsatellite markers are also potential markers for sexing^30,31^.

In this study, primers for the universal SRY and ZF markers, and six Y-chromosome-specific microsatellite markers isolated from the horse (*Equus caballus*), were tested for amplification in the Malayan tapir in an attempt to obtain functional sex-typing markers for this species. A preliminary assessment on the Malayan tapir sex ratio in the wild was also carried out using data from the Wildlife Genomic Resource Bank (WGRB) database of the Malaysian Department of Wildlife and National Parks (PERHILITAN), zoos and conservation centres, as well as from the results of molecular sexing of Malayan tapir samples from the WGRB.

## Results

### Equine Y-chromosome-specific microsatellite markers

The six equine Y-chromosome-specific microsatellite markers were expected to be amplified in male tapirs alone. Nonetheless, all the markers were either not specific to males, or yielded unspecific products (Supplementary Table S1). These markers were therefore excluded from further tests.

### SRY and ZF nucleotide sequences

DNA sequencing revealed that the amplified lengths of SRY (accession no. MN786408) and ZFX (accession no. MN786409) were 224 bp and 447 bp respectively in the Malayan tapir. Comparisons of the SRY sequence against the National Center for Biotechnology Information (NCBI) nucleotide collection of Tapiridae (taxid: 9799) did not return any results, but matches to a partial SRY region of *Equus* spp. were returned when it was compared against Perissodactyla (taxid: 9787). Comparing the ZFX sequence against the Tapiridae nucleotide collection returned only partial sequences (48 bp) that overlapped with the *T. indicus* ZFX partial coding sequence (204 bp; accession no. AY012084.1^15^).

### Validation and characterisation of sex markers

**Figure 1:**
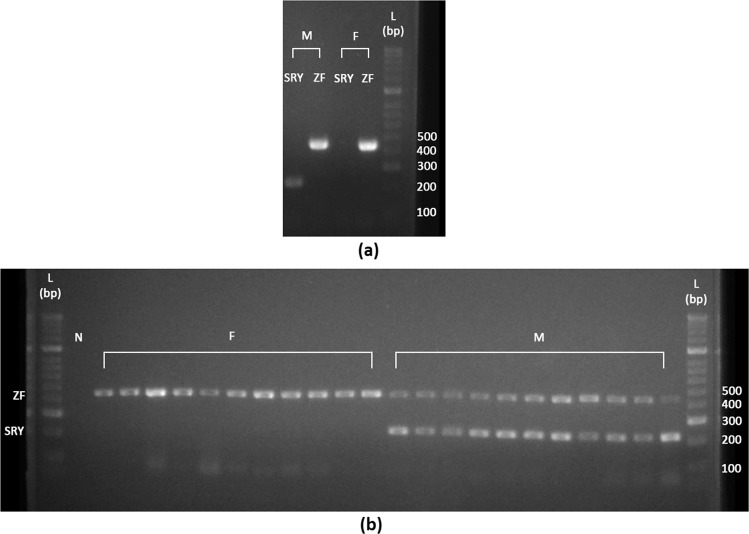
Amplification of the sex-identification region Y (SRY) and zinc finger (ZF) genes in 22 Malayan tapir individuals. (**a**) Male tapirs yielded amplicons of SRY at ~200 bp and ZFX/ZFY at ~450 bp. Female tapirs yielded only amplicons of ZFX at ~450 bp. ZF gene acts as a positive control for the PCR. (**b**) Sex-identification in 11 females and 11 males by multiplexing the primers of SRY and ZF genes in PCR, and the products were visualised on 2% agarose gel. All males and females show consistent sex-specific banding patterns. L – 50 bp ladder, N – negative control, M – male, F – female. Both images (**a**,**b**) were cropped from the full-length gels presented in Fig. S1 in Supplementary Information.

In both singleplex and multiplex PCRs, male samples showed amplification of both SRY and ZFX/ZFY, while female samples only showed amplification of ZFX (Fig. 1). Samples from 31 tapirs of known sex, or controls, were subjected to molecular sexing that were based on band pattern on agarose gel (N = 13), peak size and pattern in fragment analysis (N = 9), or both (N = 9); and all (100%) were correctly sex-typed. Characterisation through fragment analysis revealed that the fragment lengths of ZFX/ZFY and SRY (inclusive of the 20 bp tail) were 464 bp and 244 bp, respectively, and were consistent in all the 18 samples characterised. The single amplicon size yielded for the ZF gene in both the male and female samples suggested that there was no length polymorphism between ZFX and ZFY genes.

### Testing sex markers in faecal samples

**Figure 2:**
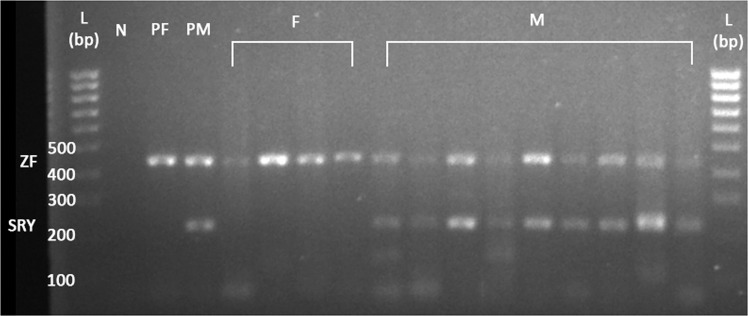
Amplification of the sex-identification region Y (SRY) and zinc finger (ZF) genes in 14 faecal samples from nine Malayan tapirs. L – 100 bp ladder, N – negative control, PF – positive control (female), PM – positive control (male), M – male, F – female. The image was cropped from the full-length gel presented in Fig. S2 in Supplementary information.

Twenty-three faecal samples were used for testing the sex markers (Supplementary Table S2). Seven faecal samples were excluded from the test due to inconsistent or unexpected sex-typing results (results not shown, but see Supplementary Table S2 for details). Three PCR replicates were prepared for 15 faecal samples and one for S20 (due to insufficient DNA sample). Thirteen samples (81.25% of the 16 samples), which expected banding pattern was observed in at least one replicate (Fig. 2), were correctly sex-typed. No amplification of SRY/ZF was obtained in all the three replicates of two of the samples. In one of the male samples, S9, only either SRY or ZF was amplified.

### Sex-typing of 18 samples with unknown sex

Sex-typing of 18 tapir samples of unknown sex in the WGRB database succeeded at a rate of 66.7% (i.e. 12 out of 18 samples; Table 1). Eight of the samples were successfully sex-typed based on the banding pattern on the agarose gel, and four samples were sex-typed by fragment analysis after producing ambiguous banding patterns on the agarose gel. The sex of a sample was accepted when at least two out of its three PCR replicates were consistent in the presence of the ZF amplicon. In the rest of the samples, which consisted of hair (n = 3), and tissues (n = 3) samples tested in fragment analysis, sex could not be determined due to inconsistent results across the three replicates, or the absence of amplification in one or both markers (Supplementary Table S3). TAP18, which sex could not be determined through DNA extracted from its hair sample, had its sex recorded in the studbook and was therefore included in the sex ratio estimation.

**Table 1:** Sex-identification of 18 Malayan tapirs of unknown sex by co-amplification of sex-determining region Y (SRY) and zinc finger (ZF) genes. Only bands or peak patterns that were consistent in two out of three trials of polymerase chain reactions were accepted for sex-typing a sample.

Sample	Sex	Source
TAP07	F	DBS
TAP12	F	DBS
TAP62	F	Tissue
TAP63	F	Tissue
TAP66	F	DBS
TAP70	M	Tissue
TAP99	M	DBS
TAP108	F	DBS
TAP17*	M	Hair
TAP18*	—	Hair
TAP19*	—	Hair
TAP20*	—	Hair
TAP21*	F	Hair
TAP22*	F	Hair
TAP38*	—	Tissue
TAP39*	—	Tissue
TAP72*	—	Tissue
TAP100*	M	DBS

M – male; F – female; DBS – dried blood spot.

*Sex-identified in fragment analysis; others were sex-identified based on banding patterns on agarose gel.

### Preliminary assessment of sex ratio

The proportion of males in a pool of wild and presumably wild tapirs (N = 66) was 0.409 (27 males:39 females), and no significant deviation from a frequency of 0.5 was found in a two-tailed binomial test at a 95% confidence interval (*p* = 0.175). When looking at wild individuals only (N = 47), the proportion of males was 0.362 (17 males:30 females), which also did not deviate significantly from parity (*p* = 0.0789).

## Discussion

### Sex-typing markers for the Malayan tapir

The sex-specific banding patterns of SRY and ZF were consistent with 100% accuracy across a total of 40 PCR reactions prepared from 31 Malayan tapir samples (nine of the samples were analysed by both agarose gel and fragment analyser, hence there were 22 PCR reactions analysed on the agarose gel and 18 on the fragment analyser). Thus, the markers are deemed reliable for sex-typing of the Malayan tapir. The negative control reaction did not yield any bands, so template contamination was unlikely. However, caution is needed to prevent human contamination since the selected primers can amplify the two DNA regions present in humans as well^32,33^.

After performing fragment analysis, the relative fragment lengths of the 18 control samples indicated that both SRY and ZF were conserved and monomorphic. The length of SRY was also reported as 224 bp in another study^34^. Monomorphism in the Malayan tapir partial ZF gene (447 bp) is similar to the horse (445 bp), unlike humans, cattle, sheep, and goats, in which ZFY and ZFX are 447 bp and 445 bp in length, respectively, when amplified using the same primer pairs^32^. It was noted that the sequence of P1-5EZ was ‘ATA TTC ACA TGG…’ from position 1 to 12, which differed from GenBank’s record of the *T. indicus* ZFX partial coding sequence, accession no. AY012084.1^15^. The deposited sequence ‘ACA CCC ACC TGG…’ is probably a more accurate sequence which has the same sequence as the human ZFX^32^.

Among the types of sample (i.e. whole blood, hair, tissue, faeces, and DBS^35^), DBS and whole blood was the easiest to sex-type—amplification success of 100%. Failure of sex-typing in half of the hair and tissue samples may be due to DNA degradation after long-term storage (at least eight years at room temperature) under simple conditions. Due to limited information, only sample TAP72 was known to be liver tissue, while TAP38 and TAP39 were unknown tissues. It is highly likely that the DNA in these tissue samples had degraded, or contained PCR inhibitors such as bile^36^. The issue of PCR failure for one or both products of SRY and ZF due to DNA degradation is also seen in the faecal samples. Since the ZF fragment to be amplified is larger than the SRY fragment, it is expected that PCR failure due to DNA degradation would affect the ZF more readily than the SRY^37^. This is in line with our observation that SRY amplicons were often obtained in male samples that had ZF amplification failures (Supplementary Table S2), even though two copies of ZF (on both the X- and Y-chromosomes) exist for every copy of SRY (on the Y-chromosome). As such, we think that the consistent presence of the SRY amplicon with or without amplification of ZF in PCR reactions may still be adequate for sex-typing a sample.

The success rate of sex-identifying the samples of unknown sex (12 successes in 18 tests, or 66.7%) was comparable to the rate (64.4%) reported in a previous study that used 49 lowland tapir scat samples^25^. However, the criteria for a successful sexing differed: in this study, consistent banding (on agarose gel) or peak patterns (on fragment analyser) from two out of three replicates were accepted for sex-typing, whereas consistency across all three replicates (on agarose gel) was required in the other study. Nevertheless, for samples that were successfully sex-identified through agarose gel banding patterns, the results were consistent in all three replicates for each of the samples.

For the faecal DNA samples, only eight out of 15 samples (that have three replicates each) were sex-typed based on the agarose gel banding patterns, if the same criteria were applied (Supplementary Table S2). We believe this sexing method to be reliable for faecal samples as all were correctly sex-typed. Noteworthy here is that we experienced contamination issues when trying to amplify the faecal DNA; the seven discarded faecal samples were suspected of possible contamination from the faeces of other species or from other tapir individuals that might have happened due to handling errors before or during the collection of faeces samples, during DNA extraction, or during PCR preparation (see Supplementary Table S2 and Supplementary Fig. S2 for more descriptions). SRY may, possibly due to contamination, be amplified in female faecal samples (less than five observations among the replicates of non-discarded samples), but often the band was extremely or comparatively much lighter compared to the male samples—which SRY and ZF bands should be more or less of equal brightness when visualised on agarose gel, as shown in Fig. 2 and Supplementary Fig. S2. In these cases, the samples were still identified as females and included in the results. Unfortunately, at the time of writing, the faecal samples were no longer available, and therefore we are not able to revalidate the results to address the contamination issues.

We recommend that future studies collect hair and faecal samples from wild and captive tapirs to further test the method’s robustness for non-invasive sexing^38^. In the context of the biodiversity and ecosystem of Peninsular Malaysia, Malayan tapir faeces are relatively easy to recognise via size and shape, so the risk of misidentifying tapir faeces is relatively low. Moreover, because the tapir is an herbivore, the issue of having another animal’s DNA in its faeces due to food intake is almost non-existent.

Finally, the success of this method in sex-typing both the lowland tapir^25^ and the Malayan tapir suggests high chances of success for other tapir species and other closely-related species. The sexing method may also be useful for future applications such as wildlife forensics and embryo implant sexing after *in vitro* fertilisation.

### Sex ratio estimation and the limitations

There has been a lack of studies on the sex ratio of Malayan tapir populations in Peninsular Malaysia. This study is the first sex ratio survey of Malayan tapir population in Peninsular Malaysia through data-mining of the WGRB Malayan tapir database and other tapir data provided by the Sungai Dusun Wildlife Conservation Centre (WCC) and zoos, and supplemented by molecular sexing tapirs of unknown sex. As shown by the binomial tests, we found no significant deviation from parity for the Malayan tapir sex ratio in Peninsular Malaysia, whether tapirs of unknown origin (but potentially sourced from the wild) were included or not. Lack of evidence for a skewed sex ratio, either in favour of males or females was also reported in a few studies of tapirs^20,39,40^. However, we acknowledge the possible contribution of small sample size to reduced statistical power.

The result of sex ratios obtained in this study is only preliminary due to: (1) a relatively small sample size (N = 66), mostly collected around 2002–2017; (2) missing or insufficient data (i.e. wild or captive-born, age, year caught, origin) and possible unidentified recaptures of individuals throughout the years without reliable means for individual identification; and (3) assumptions made on our samples in this study that could contribute to sampling bias as opposed to the assumption of random sampling, including:

(i) The tapirs used in the sex ratio estimation were treated as one population of adult tapirs regardless of actual age structure. The tapir samples were mainly collected during tapir rescue and translocation operations after receiving complaints from the public^10^. Not all incidents of tapir encounters would be recorded in the WGRB database unless biological samples were taken followed by data entry. Relevant to this, records of tapirs through these opportunistic encounters are expected to be higher in areas with higher rates of wildlife road-kill, human plantations with tapir food sources, or disturbances.
(ii) The possibility of bias in population sex ratio induced by sex-differences in behaviour, survival, and dispersal pattern that increased the probability of detecting one sex^2^ was not investigated in this study due to insufficient data. There is also a lack of studies on sex-specific philopatric behaviours in the Malayan tapir. In the lowland tapir, there was no evidence found to support such behaviour^41^. Female tapirs have a larger body size than male tapirs but whether the same is true for home range size requires more data^42,43^.

## Methods

### DNA extraction

Malayan tapir whole blood samples (n = 2) were collected from the National Zoo of Malaysia and Sungai Dusun WCC, PERHILITAN. Dried blood spots (DBS, n = 19), tissues (n = 22), and hair samples (n = 6) were obtained from the Wildlife Genomic Resource Bank (WGRB) of the National Wildlife Forensic Laboratory, PERHILITAN (permit ref. NRE 600-2/2/21 JILID 2(42)). In addition, we included 23 faecal samples collected from 11 tapirs on different dates in 2014 and 2015 at the National Zoo of Malaysia (n = 8) and Sungai Dusun (n = 15); see Supplementary Table S2 for the detailed list of samples. The samples were stored in -20 °C within two hours of collection. All the sampling procedures for the whole blood samples were approved by the Institutional Animal Care and Use Committee, Universiti Putra Malaysia (ethical approval ref.: UPM/IACUC/AUP-R033/2016). All methods were performed in accordance with the Universiti Putra Malaysia Code of Practice for the Care and Use of Animals for Scientific Purposes. Genomic DNA (gDNA) was extracted using the QIAamp^®^ DNA Mini Kit (Qiagen, Germany) following the manufacturer’s spin protocol for all the samples except the faeces, from which the gDNA was extracted using the E.Z.N.A^®^ Soil DNA Kit (Omega Bio-tek, USA). Of the 72 samples, 54 samples including 23 faecal samples with information on their sexes were used for marker testing, validation, and characterisation (see Supplementary Tables S2–S4 for details), while the other 18 samples of unknown sex were sex-typed (Table 1) to supplement sex ratio estimation.

### Cross-amplification of *Equus* sp. Y-chromosome specific microsatellite markers

The six Y-specific microsatellite markers isolated from *Equus* sp., namely: Eca.YM2, Eca.YJ10, Eca.YH12, Eca.YE1, Eca.YP9 and Eca.YA16^30^, were tested for cross-amplification in four samples (2 males: 2 females). Singleplex PCR reactions were carried out in total volumes of 10 μL, each containing 1 × MyTaq Red Mix (Bioline, Germany), 10 ng of gDNA, and 0.5, 0.8 or 1.0 μM of each primer. The PCR was run using touchdown protocols, which included an initial denaturation at 95 °C for 1 min followed by 35 cycles of denaturation at 95 °C for 15 s, annealing at 59 → 49 °C (Eca.YM2), 61 → 51 °C (Eca.YJ10), 62 → 52 °C (Eca.YH12 and Eca.YE1), 58 → 47 °C (Eca.YP9), or 54 → 45 °C (Eca.YA16) for 15 s (−1 °C per cycle), and extension at 72 °C for 15 s, ending with a single cycle of final extension at 72 °C for 3 mins.

### PCR amplification and characterisation of SRY and ZF genes

The primers for the amplification of the ZF gene (P1-5EZ: 5′-ATA ATC ACA TGG AGA GCC ACA AGC T-3′ and P2-3EZ: 5′-GCA CTT CTT TGG TAT CTG AGA AAG T-3′) and the SRY gene (Y53-3C: 5′-CCC ATG AAC GCA TTC ATT GTG TGG-3′ and Y53-3D: 5′-ATT TTA GCC TTC CGA CGA GGT CGA TA-3′) were taken from published literature^32,33^. Singleplex PCR reactions were performed in 10 μL mixture containing 1 × MyTaq Red Mix, 0.5 μM of P1-5EZ/P2-3EZ or Y53-3C/3D primer mix, and 2 ng of gDNA. The PCR was run using the touchdown profile: an initial denaturation at 95 °C for 3 mins followed by 40 cycles of denaturation at 95 °C for 20 s, annealing at 65 → 55 for 20 s (-1 °C per cycle) and extension at 72 °C for 20 s, and a final extension at 72 °C for 7 mins. For sex-typing, both SRY and ZF gene amplicons were expected in male samples, while only ZF gene amplicons were expected in female samples. The amplicons of SRY and ZFX from a male and a female respectively, were cloned into pGEM®-T Easy Vector (Promega, USA) following the manufacturer’s instructions and then sequenced to verify the amplified loci. Sequencing was run in both directions. The sequences were then searched against NCBI database using the BLAST algorithm (https://blast.ncbi.nlm.nih.gov/Blast.cgi).

The sex-typing primers were evaluated by PCR amplification in 22 samples (11 males: 11 females) consisting of tissue, whole blood, and DBS samples (Supplementary Table S4). A negative control without DNA template was included. The conditions of the PCR reactions remained the same for SRY/ZF method, but primers were multiplexed at 0.4 μM of P1-5EZ/P2-3EZ and 0.5 μM Y53-3C/3D. All the amplicons were electrophoresed through 2% agarose gel stained with RedSafe^TM^ Nucleic Acid Staining Solution (20 ml gel: 1 μl staining solution), and the gel images were captured in an ENDURO^TM^ GDS-1302 gel documentation system (Edison, NJ, USA).

Marker characterisation was carried out by modifying the primers using the method described by Vartia *et al*.^44^. The 5′-ends of P1-5EZ and Y53-3D were extended with the sequence of a third tail ‘Neomycin rev’ labelled with *6-FAM* (5′6-*FAM*-AGG TGA GAT GAC AGG AGA TC-3′). The primer concentrations of the two markers were 0.4 μM for ZF and 0.5 μM for SRY. However, the ratio of the unmodified primer:tailed primer:Neomycin rev was adjusted to 4:1:4. PCRs were run using the same touchdown protocol for 18 samples (9 males:9 females), consisting of tissue, whole blood and DBS, that were chosen from different locations to look for possible length polymorphisms (Supplementary Table S5). Fragment analysis was performed on an ABI Genetic Analyzer ABI3730XL (Applied Biosystems, USA) using LIZ500 as the size standard. The peaks were examined and scored in Peak Scanner v2.0 (Applied Biosystems, Carlsbad, CA).

Since faeces is one of the most common non-invasive sources of DNA collected for wildlife species, the molecular sexing method was also tested in 23 samples of tapir faecal DNA following the same PCR profile using the unmodified primer pairs. Three PCR replicates were prepared per sample for sex-typing and evaluation.

### Sex-typing samples of unknown sex

The 18 samples without information on their sexes were sex-typed either on 2% agarose gel, or by fragment analysis following the respective conditions described above, and in three PCR replicates per sample (Supplementary S3). Fragment analysis, a more sensitive assay, was only employed if the initial results on agarose gel were doubted due to the low quality and quantity of DNA samples that resulted in smearing, and low or no amplification. Fragment analysis results were used as a reference for sexing. In either method, band or peak patterns obtained in at least two out of three replicates and within the premise of successful amplification of ZF gene were accepted for sex-typing.

### Preliminary assessment of sex ratio

In this study, sex ratio is defined as the proportion of male, expressed as male/(male + female), throughout the text and is denoted as a value between 0 (all females) and 1 (all males). For sex ratio estimation, tapir data was extracted from separate datasheets provided by National Wildlife Forensic Laboratory, PERHILITAN (WGRB Perissodactyla database), and Sungai Dusun WCC, PERHILITAN, and supplemented by information provided by National Zoo of Malaysia and Taiping Zoo. The WGRB database contained sex information of tapirs whose samples were collected and stored in the bank. The result of molecular sexing for samples with missing sex information described above were also used to supplement the dataset. Tapirs labelled as “wild” were tapirs encountered in the wild, including the rescued, translocated, trapped, and road-killed tapirs. Tapirs of unknown origin were those neither labelled as “wild” nor “captive”. Potential replicate entries from the same individuals were identified and merged using one or multiple identifiers in combination, including name, reference number, studbook number, microchip identification number, and descriptions in the “remarks” column. Captive-born tapirs and tapirs without both information on their sexes and biological samples for sexing were omitted.

An overall estimation of sex ratio was calculated in wild tapirs pooled with samples of unknown origin but presumably wild (in total N = 66, see Supplementary Table S6), and in wild tapirs alone (N = 47). Since sex, under normal conditions, has only two outcomes: male and female, the binomial test was used as the significant test for deviation from 1:1 ratio or a frequency of 0.5, carried out using the function *binom.test* in R.3.5.1^45^ run in RStudio 1.1.447^46^.

## Supplementary information

Supplementary Information.

## Data Availability

Data analysed during this study are included in this published article (and its Supplementary Information File).
