# Supplementary material for: Molecular sexing and preliminary assessment of population sex ratio of the endangered Malayan tapir (Tapirus indicus) in Peninsular Malaysia
Source: Sci Rep. 2020 Mar 4;10:3973. doi: 10.1038/s41598-020-60552-y (PMC7055354; doi:10.1038/s41598-020-60552-y)
Supplement: Supplementary file 1 — Supplementary Information. [file 41598_2020_60552_MOESM1_ESM.docx]

**Molecular sexing and preliminary assessment of population sex ratio of the endangered Malayan tapir (*Tapirus indicus*) in Peninsular Malaysia**

Qi Luan Lim^1,2^, Yoeng Leh Tan^1^, Wei Lun Ng^3^, Christina Seok Yien Yong^1^, Ahmad Ismail^1^, Jeffrine J. Rovie-Ryan^4,5^, Norsyamimi Rosli^4^, Geetha Annavi*^1^

*^1^Department of Biology, Faculty of Science, Universiti Putra Malaysia, Selangor, Malaysia*

*^2^Wildlife Research Center, Kyoto University, Kyoto, Japan*

*^3^China-ASEAN College of Marine Sciences, Xiamen University Malaysia, Selangor, Malaysia*

*^4^National Wildlife Forensic Laboratory, Ex-Situ Conservation Division, Department of Wildlife and National Parks, Kuala Lumpur, Malaysia*

*^5^Institute of Tropical Biodiversity and Sustainable Development, Universiti Malaysia Terengganu, Terengganu, Malaysia*

*Corresponding author’s e-mail: geetha@upm.edu.my

**Supplementary Table**

**Table S1.** Six Y-chromosome specific microsatellite markers (Wallner et al. 2004) amplified under different primer concentration (0.5, 0.8, 1.0 μM) and sexes (Male, Female). √ - Presence of amplicon. × - No amplification. MB – Multiple bands.

| **Marker** | **Male** | | | **Female** | | | **Fragment Sizes (approximation)** |
| --- | --- | --- | --- | --- | --- | --- | --- |
|  | **0.5 μM** | **0.8 μM** | **1.0 μM** | **0.5 μM** | **0.8 μM** | **1.0 μM** |  |
| **Eca.YM2** | × | × | × | × | × | × | N/A |
| **Eca.YP9** | × | √ | √ | × | √ | √ | 280 |
| **Eca.YH12** | √ | √ MB | √ MB | √ | √ MB | √ MB | 100-900 |
| **Eca.YE1** | × | × | √ MB | × | × | √ MB | 350, 600, 800 |
| **Eca.YJ10** | √ MB | √ MB | √ MB | √ MB | √ MB | √ MB | 200-1000+ |
| **Eca.YA16** | × | × | √ MB | × | √ MB | √ MB | 100-400 |

**Table S2.** Testing the sex-determining region Y (SRY)/zinc finger (ZF) sex-identification method with 16 faecal samples collected from nine Malayan tapirs at Sungai Dusun Wildlife Conservation Center (SDWCC) and National Zoo of Malaysia (NZM).

| **No.** | **Sample Code** | **Sample Name^a^** | **Sex** | **Collection Date** | **Site** | **PCR Replicate^b^** | | | **Identified Sex** |
| --- | --- | --- | --- | --- | --- | --- | --- | --- | --- |
|  |  |  |  |  |  | **1** | **2** | **3** |  |
| **1.** | **S13** | Bendul | F | 14.11.2014 | SDWCC | F | F | F | F |
| **2.** | **S114** | Bendul | F | 04.11.2015 | SDWCC | F | F | F | F |
| **3.** | **S15** | Boy | M | 14.11.2014 | SDWCC | M | - | M | M |
| **4.** | **S113** | Boy | M | 04.11.2015 | SDWCC | - | - | - | - |
| **5.** | **S18** | Im | M | 14.11.2014 | SDWCC | M | M | M | M |
| **6.** | **S117** | Im | M | 04.11.2015 | SDWCC | - | M | - | M |
| **7.** | **S16** | Junior | M | 14.11.2014 | SDWCC | M | M | S | M |
| **8.** | **S111** | Junior | M | 04.11.2015 | SDWCC | S | M | M | M |
| **9.** | **S20^c^** | Lerek | F | 14.11.2014 | NZM | F |  |  | F |
| **10.** | **S118** | Lerek | F | 04.11.2015 | SDWCC | - | - | - | - |
| **11.** | **S14** | Mala | F | 14.11.2014 | SDWCC | F | F | F | F |
| **12.** | **S9** | Muharam | M | 14.09.2015 | NZM | S | S | Z | - |
| **13.** | **S119** | Muharam | M | 24.11.2015 | NZM | M | S | S | M |
| **14.** | **S131** | Muharam | M | 23.11.2015 | NZM | S | S | M | M |
| **15.** | **S115** | Semenyih | M | 04.11.2015 | SDWCC | M | M | M | M |
| **16.** | **S86** | Tuah | M | 24.11.2015 | NZM | M | S | S | M |
| **Discarded samples** | | | | | | | | | |
| **17.** | **S17** | Pradong^d^ | F | 14.11.2014 | SDWCC | S | M | - | M |
| **18.** | **S116** | Pradong^d^ | F | 04.11.2015 | SDWCC | F | - | M | - |
| **19.** | **S19** | Bertam | F | 14.11.2014 | SDWCC | M | M | S | M |
| **20.** | **S112** | Bertam | F | 04.11.2015 | SDWCC | F | M | F | F |
| **21.** | **S29** | Muharam | M | 19.10.2015 | NZM | M# | M# | M# | - |
| **22.** | **S77** | Muharam | M | 06.11.2015 | NZM | S# | S# | S# | - |
| **23.** | **S79** | Muharam | M | 03.11.2015 | NZM | S# | S# | S# | - |

^a^ According to the names of individual tapirs, some of these samples were sourced from the same tapirs, whose alternative samples (dried blood spot) according to PERHILITAN lab reference number were TAP83 (Boy), TAP94 (Bendul), TAP105 (Pradong), TAP106 (Im), and TAP107 (Semenyih). With the exception of TAP106, these dried blood spot samples were used as control samples for sex marker validation.

^b^ The codes used to indicate the results for the PCR replicates were: M – when both bands of ZF and SRY of more or less equal brightness were shown on agarose gel. F – when only a strong ZF band was shown (or when SRY is extremely faint in the presence of a strong ZF band that may be resulted from contamination). S – when only an SRY band was shown in a male sample. Z – when only a ZF band was shown in a male sample. # – when a band near 500 bp appear above the ZF band, with or without the presence of the ZF band.

^c^ Only one PCR was run for S20 due to insufficient DNA sample.

^d^ Dried blood spot of sample TAP105 was available for Pradong (here S17 and S116) and was used as one of the samples for sex marker validation. Pradong is a female tapir according to studbook, and was also identified as a female by molecular sexing method (using the DBS sample).

**Table S3.** List of 18 Malayan tapirs that were of unknown sex. √ - Presence of amplicon. × - No amplification. Numbers under the headings of three trials denote the detected fragment size of the gene. DBS – dried blood spot. ZF – Zinc finger gene. SRY – sex-determining region Y. M – male. F – female.

| **Sample** | **Source** | **Identified Sex** | **1^st^ trial** | | **2^nd^ trial** | | **3^rd^ trial** | |
| --- | --- | --- | --- | --- | --- | --- | --- | --- |
|  |  |  | **ZF** | **SRY** | **ZF** | **SRY** | **ZF** | **SRY** |
| **TAP07** | DBS | F | √ | × | √ | × | √ | × |
| **TAP12** | DBS | F | √ | × | √ | × | √ | × |
| **TAP62** | Tissue | F | √ | × | √ | × | √ | × |
| **TAP63** | Tissue | F | √ | × | √ | × | √ | × |
| **TAP66** | DBS | F | √ | × | √ | × | √ | × |
| **TAP70** | Tissue | M | √ | √ | √ | √ | √ | √ |
| **TAP99** | DBS | M | √ | √ | √ | √ | √ | √ |
| **TAP108** | DBS | F | √ | × | √ | × | √ | × |
| **TAP17** | Hair | M | 461 | 244 | 464 | 244 | 464 | 244 |
| **TAP18** | Hair | - | × | 244 | × | 244 | × | 244 |
| **TAP19** | Hair | - | 464, 466 | 244 | 464 | 242 | × | × |
| **TAP20** | Hair | - | × | × | 244 | × | 244 | × |
| **TAP21** | Hair | F | 464 | × | 464 | × | × | × |
| **TAP22** | Hair | F | 464 | × | 464 | × | 464 | × |
| **TAP38** | Tissue | - | × | × | × | 244 | 464 | × |
| **TAP39** | Tissue | - | × | 243 | × | × | × | × |
| **TAP72** | Tissue | - | × | × | × | × | × | × |
| **TAP100** | DBS | M | 464 | 244 | 464 | 244 | 464 | 244 |

**Table S4.** List of 31 Malayan tapir samples used for validating the sex-identification markers consisting of sex-determining region Y (SRY) and zinc finger (ZF) genes through agarose gel (AG; N = 22) and/or fragment analysis (FA; N = 18). DBS – dried blood spot. M – male. F – female.

| **Sample** | **Sex** | **Source** | **AG (N = 22)** | **FA (N = 18)** |
| --- | --- | --- | --- | --- |
| **TAP03** | M | DBS |  | YES |
| **TAP04** | F | DBS |  | YES |
| **TAP11** | M | DBS |  | YES |
| **TAP13** | F | Tissue | YES | YES |
| **TAP15** | M | DBS |  | YES |
| **TAP16** | M | Tissue | YES |  |
| **TAP25** | F | DBS |  | YES |
| **TAP26** | F | DBS |  | YES |
| **TAP28** | F | DBS |  | YES |
| **TAP36** | F | Tissue | YES | YES |
| **TAP37** | F | Tissue | YES | YES |
| **TAP59** | M | DBS | YES |  |
| **TAP64** | M | Tissue | YES |  |
| **TAP67** | M | Tissue | YES |  |
| **TAP68** | F | Tissue | YES |  |
| **TAP69** | F | Tissue | YES |  |
| **TAP71** | F | Tissue | YES | YES |
| **TAP74** | F | Tissue | YES |  |
| **TAP80** | M | Blood | YES | YES |
| **TAP83** | M | DBS |  | YES |
| **TAP84** | F | Tissue | YES |  |
| **TAP87** | F | Tissue | YES |  |
| **TAP88** | M | Tissue | YES |  |
| **TAP91** | M | Tissue | YES | YES |
| **TAP93** | M | Tissue | YES | YES |
| **TAP94** | F | DBS | YES |  |
| **TAP95** | F | DBS |  | YES |
| **TAP96** | M | Tissue | YES | YES |
| **TAP105** | M | DBS | YES | YES |
| **TAP107** | F | DBS | YES |  |
| **Tuah** | M | Blood | YES |  |

**Table S5.** Sample list of nine male and nine female Malayan tapirs from different locations of Peninsular Malaysia and including three samples from Singapore and two from unknown origin. M – male. F- female. DBS – dried blood spot.

| **Sample** | **Sex** | **Origin/Location** | **Sample Type** | **Captive/Wild** |
| --- | --- | --- | --- | --- |
| **TAP15** | M | Johor | DBS | Captive |
| **TAP03** | M | Singapore | DBS | Captive |
| **TAP80** | M | Singapore Zoo | Blood | Captive |
| **TAP26** | F | Johor | DBS | Wild |
| **TAP28** | F | Kedah | DBS | Wild |
| **TAP36** | F | Kelantan | Tissue | Wild |
| **TAP37** | F | Pahang | Tissue | Wild |
| **TAP71** | F | Pahang | Tissue | Wild |
| **TAP91** | M | Pahang | Tissue | Wild |
| **TAP93** | M | Pahang | Tissue | Wild |
| **TAP13** | F | Perak | Tissue | Wild |
| **TAP04** | F | Selangor | DBS | Wild |
| **TAP25** | F | Selangor | DBS | Wild |
| **TAP96** | M | Selangor | Tissue | Wild |
| **TAP105** | M | Selangor | DBS | Wild |
| **TAP11** | M | Terengganu | DBS | Wild |
| **TAP83** | M | (Johor) | DBS | Unknown |
| **TAP95** | F | (Terengganu) | DBS | Unknown |

* Locations in parentheses were deduced based on the samples name: Rengit for TAP83, which is a town name in the Johor state; and Dungun for TAP95, which is a coastal district name in the Terengganu state.

**Table S6**. List of 66 Malayan tapirs, consisting of wild (N = 47) and unknown-origin (N = 19) used for a preliminary estimation of sex ratio. Male (M) and female (F) with red font are sex of the tapirs that were identified using sex-identification markers consisting of sex-determining region Y (SRY) and zinc finger (ZF) gene. Lab reference no. is the sample code used in the Wildlife Genetic Resource Bank (WGRB) database of the Department of Wildlife and National Parks (PERHILITAN). The tapir information was retrieved from WGRB database and supplemented by information provided by Sungai Dusun Wildlife Conservation Center, Taiping Zoo, and National Zoo of Malaysia. Blank cells indicate missing or unavailable information.

| **No.** | **Name** | **Sex** | **Lab Reference No.** | **Year Caught** | **Year Collected** | **Origin/Locality** | **Wild/Unknown** |
| --- | --- | --- | --- | --- | --- | --- | --- |
|  | Tanjung Karang | F | TAP02 |  | 2005 | Tanjung Karang, Selangor | Wild |
|  | Sungai Besar | F | TAP04 | 2002 | 2005 | Sungai Besar, Selangor | Wild |
|  | Gerai | F | TAP07 |  | 2006 | Kampung Gerai, Terengganu | Wild |
|  | Gombak | F | TAP08 | 2004 | 2006 | Gombak, Selangor | Wild |
|  | Ahadiah | F | TAP10 |  | 2005 | Selangor | Wild |
|  | Kemat | M | TAP11 | 2006 | 2007 | Kampung Kemat, Terengganu | Wild |
|  |  | F | TAP13 |  | 2007 | Bruas, Perak | Wild |
|  | Fajar | F | TAP14 | 2006 | 2007 | Kampung Fajar, Sungai Tengi Selatan, Selangor | Wild |
|  |  | M | TAP16 |  | 2007 |  | Wild |
|  | Joe | M* | TAP18 | 2002 |  | Johor | Wild |
|  | Tina | F | TAP21 | 1996^a^ |  |  | Wild |
|  | Nas | F | TAP22 | 2002 |  | Johor | Wild |
|  |  | F | TAP23 |  | 2007 | Kulai, Johor | Wild |
|  | Sumbing | F | TAP25 | 2005 | 2007 | Bukit Cerakah, Selangor | Wild |
|  | Kulai | F | TAP26 | 2004 | 2007 | Kulai, Johor | Wild |
|  | Perah | F | TAP28 |  | 2007 | Kampung Perah, Kedah | Wild |
|  | Rompin | F | TAP32 | 2007 | 2007 | Rompin, Pahang | Wild |
|  |  | M | TAP33 |  | 2008 | Kampung Langkap, Negeri Sembilan | Wild |
|  |  | F | TAP34 |  | 2008 | Sungai Dusun, Selangor | Wild |
|  |  | F | TAP35 |  | 2008 | Lanchang, Pahang | Wild |
|  |  | F | TAP36 |  | 2008 | Kelantan | Wild |
|  |  | F | TAP37 |  | 2008 | Gambang, Pahang | Wild |
|  | PIAI | F | TAP56 |  | 2008 | Teluk Piai, Kuala Selangor | Wild |
|  | Rompin | M | TAP57 |  | 2008 | Rompin, Pahang | Wild |
|  |  | F | TAP58 |  | 2009 | Krau Wildlife Reserve, Pahang | Wild |
|  |  | M | TAP59 |  |  | Mentakab, Pahang | Wild |
|  |  | M | TAP67 |  | 2008 | Karak, Pahang | Wild |
|  |  | M | TAP70 |  | 2008 | Mengkarang, Pahang | Wild |
|  | Khai | F | TAP71 |  | 2009 | Rompin, Pahang | Wild |
|  | Lesong | F | TAP76 |  |  | Kemaman, Terengganu | Wild |
|  | Wan Bulan | M | TAP85 |  |  | Wan Bulan, Krau, Pahang | Wild |
|  |  | M | TAP91 |  | 2011 | Sungai Sol, Pahang | Wild |
|  | Ramsar | M | TAP93 |  | 2012 | Tapak Ramsar Tasik Bera, Pahang | Wild |
|  | Bendul | F | TAP94 | 2011 | 2013 | Ulu Bendul, Negeri Sembilan | Wild |
|  | Arang | M | TAP96 |  | 2013 | Batu arang, Kuala Selangor, Selangor | Wild |
|  | Syawal | M | TAP97 |  | 2013 | Kampung Purun, Tanjung Ipoh, Negeri Sembilan | Wild |
|  | Cherating | F | TAP98 |  | 2013 | Pantai Cherating, Pahang | Wild |
|  |  | M | TAP99 |  | 2013 | Sri Menanti, Negeri Sembilan | Wild |
|  | Oden | M | TAP101 |  | 2014 | Kampung Mertang, Seri Menanti, Negeri Sembilan | Wild |
|  | Titi | F | TAP102 |  | 2014 | Kampung Felda Titi, Jelebu, Negeri Sembilan | Wild |
|  | Gementah | M | TAP104 |  | 2017 | Gementah, Johor | Wild |
|  | Semenyih | M | TAP105 |  | 2015 | Semenyih, Selangor | Wild |
|  | Pradong | F | TAP107 | 2010 | 2016 | Jelebu, Negeri Sembilan | Wild |
|  | Emerald | F | TAP108 |  |  | Emerald West, Sungai Bakau, Rawang | Wild |
|  | Kampar | F | TAP109 |  |  | Kampung Kampar, Kuala Klawang, Jempol, Negeri Sembilan | Wild |
|  | Lerek | F |  | 2014 |  | Kampung Air Lerek, Negeri Sembilan | Wild |
|  | Tissy | F |  | 2003^a^ |  |  | Wild |
|  |  | M | TAP05/TPR9 |  | 2007 |  | Unknown |
|  |  | M | TAP06/TPR1 |  | 2004 |  | Unknown |
|  |  | M | TAP09/TPR4 |  | 2004 |  | Unknown |
|  |  | F | TAP12 |  |  | Bangas^b^ | Unknown |
|  | Grik^b^ | M | TAP17 |  |  |  | Unknown |
|  |  | F | TAP62 |  |  |  | Unknown |
|  |  | F | TAP63 |  |  |  | Unknown |
|  |  | M | TAP64 |  | 2007 |  | Unknown |
|  |  | F | TAP68 |  | 2007 |  | Unknown |
|  |  | F | TAP69 |  | 2007 |  | Unknown |
|  |  | F | TAP73 |  | 2008 |  | Unknown |
|  |  | F | TAP74 |  | 2008 | Meru KM41??? ^b^ | Unknown |
|  |  | M | TAP75 |  | 2011 | Krau Wildlife Reserve | Unknown |
|  | Rengit^b^ | M | TAP83 |  | 2010 |  | Unknown |
|  | Anak Kulai | M | TAP88 |  | 2010 |  | Unknown |
|  | Ramadani | F | TAP92 |  | 2011 | Krau Wildlife Reserve, Pahang^b^ | Unknown |
|  |  | F | TAP95 |  | 2012 | Dungun^b^ | Unknown |
|  | Purun^b^ | M | TAP100 |  | 2014 |  | Unknown |
|  | Bagas | M | TAP110 |  | 2017 | Krau Wildlife Reserve Institute of Biodiversity, Pahang^b^ | Unknown |

^a^ estimated year

^b^ Potential names of locations where the Malayan tapir individuals were found. Tapirs were sometimes named after the location they were found.

* Identified from the Taiping Zoo studbook.

**Supplementary Figure**

**
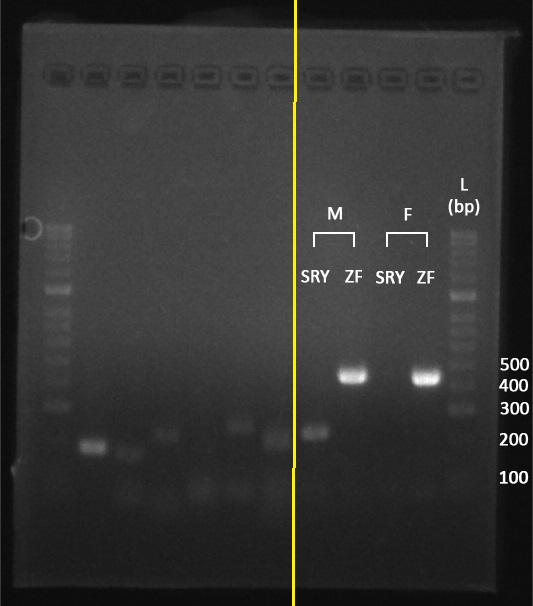
**

**(a)**

**
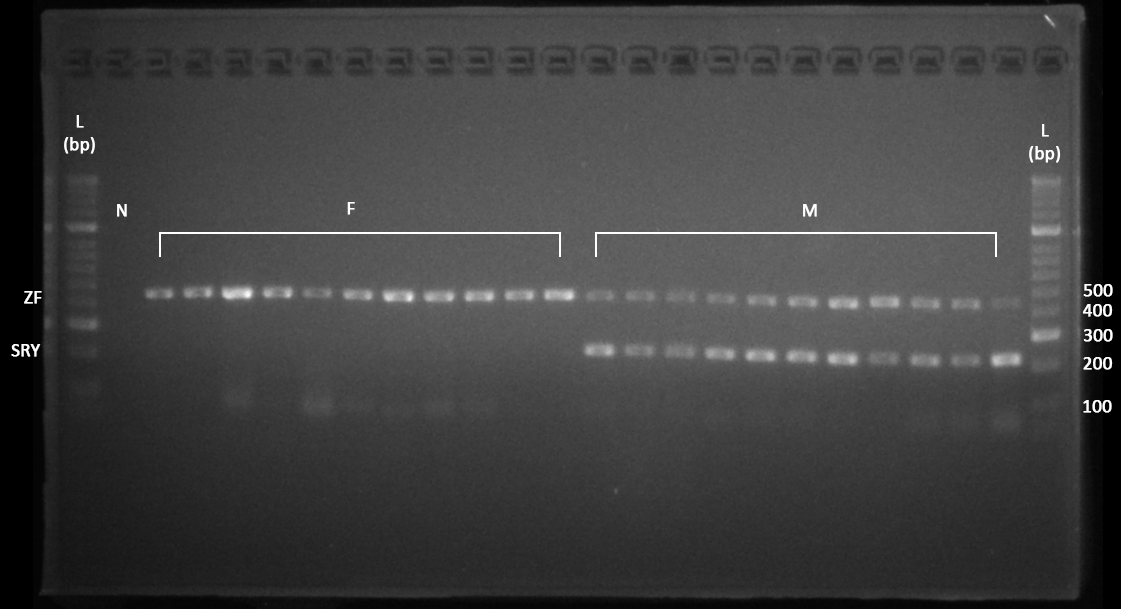
**

**(b)**

**Fig. S1**. Full-length images of the cropped gels presented in Fig. 1 in the main text. (a) Singleplex polymerase chain reaction (PCR) amplification of sex-determining region Y (SRY) and zinc finger (ZF) gene in one Malayan tapir sample (right side of the yellow line). (b) Multiplex PCR of the two genes in 22 Malayan tapir samples. L – 50 bp ladder, N – negative control, M – male, F – female.

**
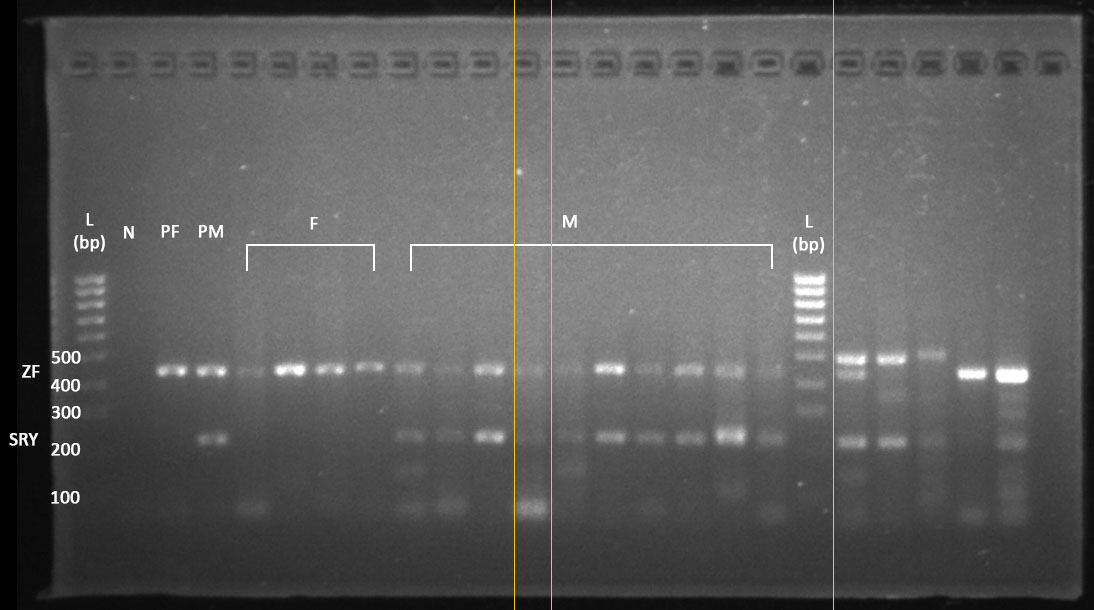
**

**Fig. S2.** Full length image of the cropped gel presented in Fig. 2 (first and third boxes, from left to right, of the gel were merged). Amplification of the sex-identification region Y (SRY) and zinc finger (ZF) genes in 13 faecal samples from nine Malayan tapirs. L – 100 bp ladder, N – negative control, PF – positive control (female), PM – positive control (male), M – male, F – female. Second box shows the result of one of the replicates of a male sample (second male sample from left) shown in gel. The last box on the right shows examples of the discarded samples yielding extra bands (~500 bp), and the contradicting results between replicates (the last two columns) of one discarded female sample that yielded SRY band and unspecific bands in one of the replicates.

**Supplementary Note: Sequences of SRY and ZFX**

>TAP93_SRY (224 bp)

CCCATGAACGCATTCATTGTGTGGTCTCGTGATCACAGGCGCAAGGTGGCTCTTGAGAATCCCCAAATGCGAAACTCAGAGATCAGCAAGTGGCTGGGATGCCAGTGGAAAAAGCTTACGGAAGCGGAAAAATGGCCATTCTTCGAGGAGGCAGAGAGACTACGGGCCGTGCACCGAGAGAAATACCCGGACTATAAATATCGACCTCGTCGGAAGGCTAAAAT

>TAP13_ZFX (447 bp)

ATATTCACATGGAGAGCCACAAGCTGACCAGCAAGGCAGAGAAGGCCATCGAATGTGATGAGTGTGGGAAGCATTTCTCTCATGCTGGGGCTTTGTTTACTCACAAAATGGTGCATAAGGAGAAAGGAGCCAACAAAATGCACAAGTGTAAATTCTGTGAATATGAGACAGCTGAACAAGGGTTGTTGAATCGCCACCTTTTGGCGGTCCACAGCAAGAACTTTCCTCATATTTGTGTGGAGTGCGGTAAAGGTTTTCGGCACCCATCAGAGCTCAAAAAGCACATGCGAATCCATACTGGGGAGAAGCCGTACCAATGCCAGTACTGCGAGTATAGGTCTGCAGACTCTTCTAACTTGAAAACGCATGTAAAAACTAAGCATAGTAAAGAGATGCCATTCAAGTGTGACATCTGTCTTCTGACTTTCTCAGATACCAAAGAAGTGC

**References**

Wallner, B., Piumi, F., Brem, G., Müller, M. & Achmann, R. Isolation of Y chromosome-specific microsatellites in the horse and cross-species amplification in the genus *Equus*. *J. Hered.* **95**, 158–164 (2004).
